# Failed Attempt to Recommend Noise Cancelling Headphones for Knee Arthroplasty Surgeons—Results of a Pilot Study

**DOI:** 10.3390/medicina59020320

**Published:** 2023-02-09

**Authors:** Christian Stadler, Matthias Luger, Bernhard Schauer, Stella Stevoska, Tobias Gotterbarm, Antonio Klasan

**Affiliations:** 1 Department for Orthopaedics and Traumatology, Med Campus III, Kepler University Hospital, Krankenhausstr. 9, 4020 Linz, Austria; 2 Johannes Kepler University Linz, Altenberger Str. 96, 4040 Linz, Austria; 3 AUVA Trauma Hospital Styria Graz, Göstinger Str. 24, 8020 Graz, Austria

**Keywords:** active noise cancelling headphones, total knee arthroplasty, noise exposure, noise induced hearing loss

## Abstract

*Background and Objectives*: Noise exposure during total knee arthroplasty (TKA) has been demonstrated to exceed thresholds that are deemed as over-exposure by industry noise level standards. With orthopedic surgeons being at risk of suffering from Noise Induced Hearing Loss, the purpose of this pilot study was to evaluate the viability of the use of industry grade active noise cancelling headphones (ANCH) during TKA. *Material and Methods:* In this prospective pilot study, 10 TKA were performed. In five of these cases, surgeon, assistant, scrub nurse and anesthetist wore ANCH with automatic noise level dependent noise attenuation above 82 dB. A validated 14-item questionnaire was used after each case to evaluate the quality of communication, performance, teamwork and mental load. In seven cases a calibrated sound level meter was used to measure the operating theatre noise. Peak sound level (LApeak), A-weighted continuous sound level (LAeq) and A-weighted noise exposure averaged for an 8-h time-period (LEPd) were calculated. *Results:* There was no perceived benefit of ANCH for the surgeons (*p* = 0.648), assistants (*p* = 0.908) and scrub nurses (*p* = 0.251). There was an overall improvement observed by anesthetists (*p* = 0.001). A worse communication while wearing ANCH was reported by surgeons but not by the rest of the team. Average LApeak was 90.6 ± 3.2 dB(C), LAeq was 61.9 ± 1.0 dB(A) and LEPd was 53.2 ± 1.2 dB(A). *Conclusions:* Industry grade ANCH seem to provide no benefit for surgeons, assistants and scrub nurses during TKA, while anesthesiologists seem to benefit from the use of ANCH during TKA. Due to the limitations of this pilot study, further studies with larger study populations are necessary to adequately investigate the use of ANCH during TKA.

## 1. Introduction

Orthopedic operating theatres are known to be a working environment with considerable noise exposure—especially when joint replacements such as Total Knee Arthroplasty (TKA) or Total Hip Arthroplasty (THA) are performed [1,2]. In particular the use of instruments such as saws for bone preparation and hammers for chiseling of osteophytes or component placement is associated with substantial noise exposure. Hammering during TKA is reported to produce sound levels greater than 100 dB(A) and the use of oscillating saws during TKA is reported to reach sound levels greater than 95 dB(A) [2]. These noise exposures have been proven to be close to or in some cases even exceed certain recommended noise levels recommended by different organizations such as the National Institute for Occupational Safety and Health in the United States (U.S.) or the Health and Safety Executive in the United Kingdom (U.K.), which recommends a safety threshold regarding noise exposure of 85 dB(A) for an 8 h period [3,4,5]. However, high noise exposures are not only present in orthopedic operating theatres but also in operating theatres of several other surgical specialties [6,7,8].

Noise in general is not only compromising concentration and communication but is also possibly leading to hearing loss when exceeding a certain exposure level over a certain time, which represents a serious hazard in health care facilities and especially in operating theatres [1,9,10,11,12,13,14]. Health personnel and especially surgical orthopedic staff are at risk for suffering from Noise Induced Hearing Loss (NIHL), with evidence of NIHL found in up to 50% of orthopedic surgeons [15]. NIHL in general seems to become more and more frequent within the general population with a predicted significant increase in patients suffering from NIHL in the U.K. until 2035 [16]. Moreover, an increasing number of NIHL claims was reported in New Zealand within the last centuries leading to an substantial increase in societal costs [17]. Additionally, high noise exposures in operating theatres seem to negatively impact the performance of the surgical team as well as the communication within the surgical team, which might lead to complications for example caused by acoustical misunderstandings [18,19]. Additionally, extensive noise levels in operating theatres seem to be associated with a higher rate of postoperative complications like for example surgical site infections [20,21].

Apart from the passive noise cancelling provided by the housing of headphones, Active Noise Cancelling Headphones (ANCH) use anti-noise to suppress disruptive ambient noise. For this purpose, ANCH typically feature microphones which are directed outwardly and analyze the ambient sounds. The speakers within the headphones then generate an inverted sound, which results in those simultaneously present sound waves—the ambient sound waves and the accordingly generated inverse sound waves—cancelling each other out before they reach the tympanum of the person who is wearing the ANCH. As a result, ambient noise is reduced significantly and ideally hardly even heard by the wearer of the ANCH. The technology works especially for noise or sounds at low frequencies [22,23]. While mainly being used for recreational activities such as travelling and commuting or for industrial work, ANCH are also in use sparsely in certain areas of health care facilities such as intensive care units to reduce noise exposure of patients [24,25]. The use of ANCH has also been proven to reduce patients’ anxiety while being awake during surgery and seems to improve the overall experience during wide awake surgery as well [26,27].

The use of ANCH could potentially reduce noise exposure and improve concentration as well as communication within the surgical staff during joint replacement. However, up to date the use of ANCH or other hearing protections in operating theatres by surgeons, anesthesiologists or nurses is not common and there is hardly evidence of the effectiveness of the use of ANCH during orthopedic surgeries. Therefore, the aim of this study was to evaluate possible benefits of the use of ANCH during TKA while also analyzing the noise exposure during TKA using conventional surgical instruments.

## 2. Materials and Methods

### 2.1. Study Design and Technical Implementation

**Figure 1:**
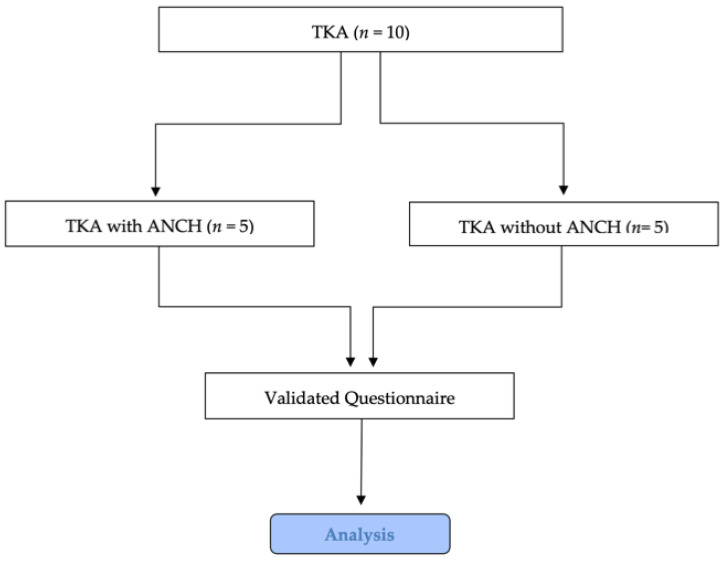
Flowchart showing the study design. TKA: Total Knee Arthroplasty; ANCH: Active Noise Cancelling Headphones.

In this prospective study a total of 10 primary TKA (Persona Knee System, Zimmer Biomet, Warsaw, IN, USA) were performed using conventional instruments. The battery driven Trauma Recon System (TRS) Modular Drive (Model 05.001.201, DePuy Synthes, Raynham, MA, USA) was used for drilling and the TRS Recon Saw (Model 05.001.204, DePuy Synthes, Raynham, MA, USA) with Prismatic Sagittal Saw Blade (De Soutter Medical Ltd., Aston Clinton, Aylesbury, UK) was used for the bone cuts. A new saw blade was used for every case. In 5 of these cases the surgical team consisting of the surgeon, the assistant, the scrub nurse as well as the anesthesiologist wore industry grade ANCH (PELTOR™ ProTac™ III, 3 M, Saint Paul, MN, USA) for the duration of the entire surgical procedure. The pilot study, including the off-label use of the headphones, has been approved by the local ethics committee. All patients provided written informed consent. The other 5 cases were performed conventionally without the use of ANCH. A detailed flowchart regarding the study design is shown in Figure 1. There was no background music in any of the cases performed in the study [28]. The industry grade ANCH used in this study feature an electronic noise level dependent active noise cancellation which is activated automatically at a noise level of 82 dB. The reported noise reduction rating (NRR) is 21 dB. Below a noise level of 82 dB the ambient sounds such as voices are reproduced electronically and transferred to the ANCH wearer’s ears which allows an undisturbed communication within the surgical team during non-noisy parts of the surgical procedure.

After the surgical procedure every study participant was handed a validated 14-item questionnaire to evaluate the quality of communication, performance, teamwork and mental load and compared between ANCH and ANCH-free cases [29,30,31]. The statements presented at the questionnaire were rated on a scale from 0 to 10 by the study participants with 0 points implying no agreement at all with the presented statement and 10 points implying full agreement with the questionnaire’s statement. For each questionnaire an overall score was calculated. The score of negative statements implying a poor communication or a high subjective noise level (e.g., “I was distracted, annoyed, stressed or bothered by the noise level in the room”) were subtracted from 10. As a result, higher scores within the questionnaires implied better communication, respectively, collaboration within the surgical team or a lower subjective noise level with 140 points being the highest possible score and 0 points being the lowest possible score. Optionally, subjective issues perceived by the user were documented at the end of the questionnaire.

Furthermore, the noise level at the operating theatre was measured during 7 procedures using a calibrated sound level meter with integrated storage (Testo 816-1, Testo SE & Co. KGaA, Tittisee-Neustadt, Germany). The sound level meter was placed consistently on a tripod behind the sterile drape next to anesthetist with the microphone of the sound level meter protruding beyond the sterile drape. Peak sound pressure (LApeak), the A-weighted continuous sound level (LAeq) and the A-weighted exposure averaged for an 8-h time period (LEPd) were recorded during those sessions.

### 2.2. Statistical Analysis

SPSS (version 27.0, IBM, Armonk, NY, USA) was used for the statistical analysis. A Kolmogorov–Smirnov test was performed to test for normal distribution. As for metric scaled data arithmetic mean value and the standard deviation were calculated. These two parameters were reported as mean ± standard deviation. Kruskal–Wallis was used for the evaluation of differences between the study groups regarding non-normally distributed parameters, and the *t*-test was used to analyze normally distributed parameters. The difference between nominally scaled parameters was analyzed using the chi square test. The level of significance was defined at *p* ≤ 0.05.

## 3. Results

The evaluation of the questionnaires revealed an overall average score of 122.6 ± 4.6 points for study participants using the ANCH and 118.5 ± 6.6 points for study participants not using the ANCH (*p* = 0.03) during TKA. The analysis of the study’s subgroups showed the following outcomes regarding the questionnaire: The average score of surgeons was 123.2 ± 2.3 with ANCH and 122.6 ± 1.7 without ANCH (*p* = 0.648), while the average score of assistants was 120.6 ± 6.3 with ANCH and 120.0 ± 9.2 without ANCH (*p* = 0.908). Nurses rated the questionnaire with an average of 123.8 ± 5.3 points after using ANCH and 120.6 ± 2.3 points without the use of ANCH (*p* = 0.251) while the mean questionnaire’s score of anesthesiologists was 122.6 ± 4.4 points with ANCH and 110.8 ± 3.2 points without ANCH (*p* = 0.001). Apart from the overall scores, surgeons, assistants, nurses and anesthetists stated to hear less clearly when wearing ANCH compared to wearing no headphones. An improvement in communication was stated from assistants, nurses and anesthetist. The overall communication was rated slightly worse by surgeons while wearing ANCH. All study groups rated their focus and concentration during surgery higher while wearing ANCH compared to wearing no headphones. Moreover, surgeons, nurses and anesthetists felt that steps took longer than necessary because they had to repeat or clarify what they were saying without the use of ANCH compared to while wearing ANCH. On average, members of every study group were subjectively more successful in performing their tasks while not wearing ANCH, except for assistants, who stated no difference regarding how successful they were in performing their tasks with or without the use of ANCH. The detailed results of the questionnaire are shown in Table 1.

**Figure 2:**
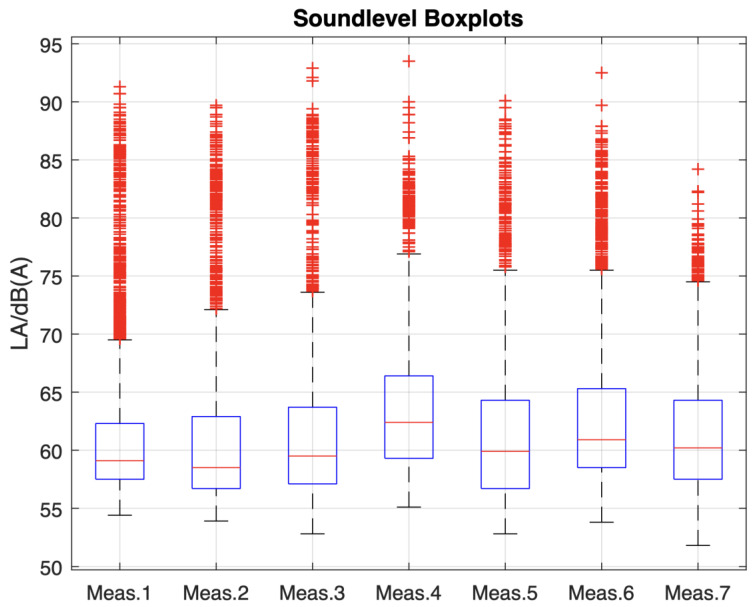
Boxplot of the sound levels in dB(A) during the investigated TKA sessions.

The noise evaluation during TKA revealed an average LApeak of 90.6 ± 3.2 dB(C) with the highest measured LApeak during the investigated TKA sessions being 93.5 dB(C). The average LAeq was 61.9 ± 1.0 dB(A) (Figure 2) while the average LEPd was 53.2 ± 1.2 dB(A) during TKA.

## 4. Discussion

The results of this study reveal no perceived benefit for the use of industry grade ANCH for surgeons, assistants and scrub nurses during TKA in overall communication, performance, teamwork and mental load. Only anesthesiologists seem to benefit from the use of ANCH during TKA, as the average questionnaire’s score after the use of ANCH was significantly higher than without the use of ANCH. Interestingly, regardless of the overall questionnaires’ scores, each of the study groups mentioned above seemed to hear less clearly while using ANCH than without. This might reflect the industrial background of the headphones used within this study, which are not specifically built for the purpose they were used for within this study as they should primarily protect the wearer from hazardous noise while at the same time being as robust as possible for example while being used on construction sites. Therefore, they are not built for the use in an operating theatre, where continuous sounds such as the surgical suction within the operating field or humming noises from other electronical medical devices generate a challenging environment for ANCH, as they should filter those sounds as good as possible while still enabling a clear communication between all members of the surgical team.

However, the overall average score of the surgeons after the use of ANCH was slightly but not significantly higher than after not using ANCH. Nevertheless, two surgeons mentioned the worry of possibly not hearing relatively silent and brief sounds during surgery that could indicate complications such as for example the cracking of a bone while fracturing, which might—if not instantly heard during surgery—not get exposed or discovered until well after completion of the surgery. Therefore, the use of any devices which possibly impair the hearing of the surgeon should be reconsidered very critically as it could result in severe complications. A clear and stable communication within the whole surgical team must be ensured if any assistive devices which possibly impair the communication are used during surgery.

The anesthesiologists appreciated the ANCH especially during phases of the procedure with high noise exposure such as bone sawing or implanting the original components during TKA with no perceived negative side effects on the overall communication. Additionally, two anesthesiologists mentioned that they liked the possibility to temporarily take off the ANCH during quieter phases of the procedure, although they were instructed not to do so prior to the start of the procedure for study purposes. However, the regular use of a hearing protection for anesthetists could possibly be implemented with less effort compared to implementing a hearing protection for surgeons as there are several factors to consider in terms of preserving the sterility of the surgical field. As the working environment of anesthetists is not sterile for most of the tasks that they have to accomplish during joint replacement, it potentially would be easier to provide a hearing protection for anesthetists that could be used only temporarily and for example could be taken off during non-noisy parts of the surgical procedure. Within this study, anesthetists mentioned that they would appreciate if a hearing protection was provided to them, which they could take on optionally and only temporarily during noise parts of a surgery.

All study groups, with the exception of the surgeons, reported an improved communication while using ANCH. With communication in the operating room being a highly complex subject it is important to improve it as best as possible as communication has been shown to affect operating theatre practices in many studies [32]. Poor communication may even lead to an increased incidence of adverse events and therefore may reduce patient safety in the operating theatre [33,34]. In the present study, one main drawback was the subjectively worse communication reported by the surgeons, who especially reported issues in communication during noise intensive parts of the surgery where the automatic noise cancelling function of the ANCH was turned on. While having an impaired quality of hearing themselves while wearing the ANCH, surgeons reported that they could not be confident that all other members of the surgical team actually could hear clearly what they were saying during noisy parts of the surgery when the automatic noise cancelling function was turned on. This might have resulted in the slightly worse rating of the overall communication of surgeons while wearing ANCH. Additionally, there is also a slight, but existing delay regarding the automatic activation and deactivation of the noise cancelling function of the ANCH, which might impair communication further. However, assistants, nurses and anesthetists rated the communication slightly better while wearing ANCH. Most of the study participants stated, that they became more aware of the importance of the communication within the surgical team while wearing ANCH and therefore tried to communicate as clear as possible. Thus, the improved communication reported by assistants, nurses and anesthetists might be study related cannot be generalized or applied to other settings, where for example a regular use of hearing protections is common. Eventually, the industry grade ANCH used within this study are designed for communication while being worn for example at constructions sites. However, they might be primarily built to be used in more open settings, with potentially less echo and less sound sources, which are producing noise simultaneously at different frequencies. Additionally, the industry grade design of the ANCH used within this study focus on a robust and sturdy design potentially slightly sacrificing wearing comfort when compared to conventional ANCH, which are designed for recreational use exclusively.

There are many factors influencing speech intelligibility and as a consequence communication in an operating room such as room size or objects placed in the operating room [35]. However, previous studies have shown significant negative effects of noise on the communication in the operating room [11,18,36]. High noise exposure in the operating room also seems to be associated with a higher rate of postoperative complications like for example higher rates of surgical site infections after hernia repairs [20]. An appropriate tool to suppress noise while still enabling an adequate communication could therefore help to improve the overall conditions for a best possible communication.

The results of this study reveal that common industry grade ANCH might not improve the subjective overall communication and collaboration within the surgical team significantly, with exception of the anesthesiologists. Specific ANCH designed for a medical use in the operating room might be better suited to improve communication within the surgical team. Anesthesiologists typically communicate less with the surgical team [37], once the surgery has started, which might explain the lack of perceived communication issues when ANCHs were worn.

Despite the issues regarding the subjective communication and quality of hearing, all study groups stated that their concentration and focus was higher while wearing ANCH compared to wearing no headphones or hearing protections. Due to the study design and the lack of questions regarding the reasons for an improved or deteriorated concentration, we are not able to comment on the reasons of this finding in detail. One possible explanation could be that the reduced level of ambient noise and sounds enabled a better concentration and focus for the person who was wearing ANCH.

As for the noise evaluation within the performed procedures, an average LApeak of 90.6 ± 3.2 dB(C) was measured with 93.5 dB(C) being the overall highest measured noise level. The average measured LAeq was 61.9 ± 1.0 dB(A) while the average LEPd was 53.2 ± 1.2 dB(A) during TKA performed within this study. Compared to other studies which also investigated noise levels during TKA, the noise exposure within this study was lower in comparison [1,38]. Especially when compared to robotic assisted TKA the noise exposure levels measured within this study were quite lower [39]. Nevertheless, the noise exposure levels are close to some of the maximum noise levels recommended by different health organizations [4].

Even if the recommended limits were not exceeded within this study, wearing a hearing protection while performing TKA should be considered as many studies have rated the noise exposures during TKA as health hazard in particular for suffering from NIHL [40]. Furthermore, also the noise levels at operating rooms of other surgical specialties could be evaluated as well, as high noise exposures are not exclusive to orthopedic operating rooms [7].

One possible approach for lowering the subjective noise exposure of the surgical staff could be music playing in the background in the operating theatre during surgery. Although music generally produces an even higher sound level in the operating theatre, a large portion of the surgical staff seems to favor music playing in the background of the operating theatre over a setting, where no music is playing [19]. Moreover, music in the operating theatre seems to affect several different tasks in several different ways. While both positive and negative effects of music on surgical tasks are reported, the positive effects seem to outweigh the negative effects when the right type of music is played at the right sound level such as for example classical music at a medium sound level, which seems to potentially enhance speed and accuracy of surgeons [41]. Additionally, aside from the surgery itself, music seems to possibly influence the turnover time in between surgeries, as fast music playing in between two surgeries was shown to significantly reduces the turnover time [42]. However, there are also studies reporting a negative effect of music playing in the operating theater as it might make repeated requests more frequent which might result in an prolongation of the operation time [28]. A possible solution for the surgeon or another surgical team member to immediately stop music in the operating room could be voice controlled speakers. Using those, no member of the surgical team would have to leave the sterile environment to temporarily pause music playback when silence is necessary for example in case of a critical event where an unimpaired communication is crucial [43].

There are several limitations to this study that must be mentioned. Due to a lack of comparative data, this study was conducted as a pilot project at a single study center with very limited study participants. Therefore, the results of this study must be interpreted with caution. The results of the study, however, do not encourage further investigation with industry grade ANCH. Potentially, the use of other conventional models of ANCH with better wearing comfort and optimized noise cancelling function—optimally developed and tuned specifically for the use in a surgical setting—could improve the overall user experience within the surgical staff. Furthermore, the tripod where the sound level meter measuring the noise exposure during TKA was mounted on was placed behind the sterile drape next to anesthetist. Therefore, the distance to the surgical field was slightly larger compared to the surgeon’s and assistant’s heads, which might have caused lower measured noise levels. In future studies noise level measuring devices could be placed closer to the heads of the surgeon or the assistant. Moreover, surgical teams varied within this study but were not randomized. Therefore, interpersonal factors between the members of each surgical team might have also influenced the atmosphere and communication during TKA. Additionally, there was no music playing in the background of the operating theatre while conducting this study. Therefore, the results and findings of this study cannot be applied to other settings, where music is playing in the operating theatre, which might influence the overall subjective perception of ambient noise during surgery. Furthermore, different types of saws and drills might produce higher or lower sound levels compared to the saws and drills that were used within this study, which represents another limitation of this study as the findings of this study are hardly applicable to other settings where other types of instruments are used. Moreover, the ambient level of noise might differ at different operating theatre settings as there are many factors influencing the ambient sound level such as ventilation systems or different types of medical devices like respirators used by the anesthetists. Lastly, only an orthopedic surgical setting was investigated within this study. Therefore, no conclusions can be drawn regarding settings in operating theatres of other surgical specialties, which also represents a substantial limitation of the present study.

## 5. Conclusions

ANCH seem to provide no benefit for the surgeon, assistant or scrub nurse during TKA. Only anesthesiologists seem to benefit from the use of ANCH during TKA. However, the use of industry grade ANCH during TKA could not be reliably determined due to the shortcomings and limitations of this pilot study.

Further studies with purpose-built ANCH for the surgical team and larger study populations would be necessary to adequately investigate the use of ANCH during TKA, although the findings of this pilot study are hardly encouraging for such an endeavor.

## Figures and Tables

**Table 1:** Detailed results of the questionnaire.

Question/Statement:	Surgeon with ANCH	Surgeon without ANCH	Assistant with ANCH	Assistant without ANCH	Nurse with ANCH	Nurse without ANCH	Anaesthesist with ANCH	Anaesthesist without ANCH
I heard clearly during the case.	9.0 ± 1.0	9.4 ± 0.5	7.8 ± 1.1	9.2 ± 1.0	6.0 ± 1.2	9.6 ± 0.5	7.8 ± 0.4	8.4 ± 0.9
I had to repeat myself because people didn’t understand/hear my message the first time.	9.0 ± 0.8	8.6 ± 0.5	9.4 ± 0.9	9.4 ± 0.5	6.4 ± 1.1	8.4 ± 0.5	6.8 ± 0.8	8.0 ± 0.0
Overall, I felt that OR team communication was?	8.2 ± 0.4	8.4 ± 0.4	8.0 ± 0.7	7.8 ± 1.1	9.2 ± 0.8	8.6 ± 0.5	8.4 ± 0.5	8.2 ± 0.4
I would grade my focus and concentration during this case.	9.8 ± 0.4	9.4 ± 0.5	9.4 ± 1.3	8.2 ± 1.6	9.8 ± 0.4	9.0 ± 0.0	10.0 ± 0.0	8.4 ± 0.9
Steps took longer than necessary because I or others had to repeat/clarify what they were saying.	8.6 ± 0.6	9.0 ± 0.2	9.0 ± 0.2	9.4 ± 0.5	9.4 ± 0.9	9.4 ± 0.5	9.0 ± 0.0	9.2 ± 0.4
Based on the team’s performance during this case, I would feel perfectly safe being treated here.	9.8 ± 0.4	8.6 ± 0.5	9.2 ± 1.1	9.2 ± 1.1	9.8 ± 0.4	9.0 ± 0.7	9.0 ± 0.0	7.2 ± 0.4
How successful were you in performing your task?	9.8 ± 0.4	9.0 ± 0.2	9.6 ± 0.9	9.0 ± 1.0	9.4 ± 0.5	9.4 ± 0.5	10.0 ± 0.0	7.4 ± 0.9
Team morale during this case was high.	9.4 ± 0.5	9.2 ± 0.4	10.0 ± 0.0	9.4 ± 0.5	9.4 ± 0.9	7.6 ± 0.5	9.0 ± 0.0	7.6 ± 0.5
I felt comfortable intervening in this procedure when I had concerns about what was occurring.	9.4 ± 0.5	9.2 ± 0.4	9.2 ± 0.4	8.4 ± 1.5	9.8 ± 0.4	8.0 ± 0.1	7.2 ± 2.0	7.0 ± 0.0
Overall, we worked efficiently as a team.	9.4 ± 0.5	8.0 ± 0.2	9.8 ± 0.4	8.8 ± 1.1	8.8 ± 1.1	8.8 ± 1.0	9.0 ± 0.2	7.8 ± 0.4
I felt fatigued/exhausted after this case.	8.6 ± 0.5	8.4 ± 0.5	8.2 ± 0.8	8.4 ± 1.5	9.6 ± 0.9	7.6 ± 0.5	10.0 ± 0.0	8.6 ± 0.5
I was irritated, stressed, or annoyed during this case.	8.2 ± 1.1	8.4 ± 0.5	7.8 ± 0.8	8.8 ± 1.1	9.4 ± 1.3	9.0 ± 0.0	9.2 ± 0.8	8.6 ± 0.5
How hard did you have to work mentally and physically to accomplish your task?	7.0 ± 1.6	8.8 ± 0.4	6.8 ± 1.1	6.8 ± 1.3	7.2 ± 0.4	8.6 ± 0.5	8.4 ± 0.5	5.6 ± 1.3
I was distracted, annoyed, stressed, or bothered by the noise level in the room.	7.0 ± 1.6	8.8 ± 0.4	6.4 ± 1.3	7.2 ± 0.8	9.6 ± 0.9	7.6 ± 0.5	8.8 ± 0.8	8.8 ± 0.4
Overall Score	123.3 ± 2.3	122.6 ± 1.7	120.6 ± 6.3	120.0 ± 9.2	123.8 ± 5.3	120.6 ± 2.3	122.6 ± 4.4	110.8 ± 3.2

## Data Availability

The data presented in this study are available on request from the corresponding author.
